# A Novel Multiplex HRM Assay to Detect Clopidogrel Resistance

**DOI:** 10.1038/s41598-017-16310-8

**Published:** 2017-11-22

**Authors:** Lichen Zhang, Xiaowei Ma, Guoling You, Xiaoqing Zhang, Qihua Fu

**Affiliations:** 1 School of Biomedical Engineering, Shanghai Jiao Tong University, Shanghai, 200127 P.R. China; 2 Department of Laboratory Medicine, Shanghai Children’s Medical Center, Shanghai Jiao Tong University School of Medicine, Shanghai, 200127 P.R. China; 3 Department of Laboratory Medicine, Renji Hospital, Shanghai Jiao Tong University School of Medicine, Shanghai, 200127 P.R. China

## Abstract

Clopidogrel is an antiplatelet medicine used to prevent blood clots in patients who have had a heart attack, stroke, or other symptoms. Variability in the clinical response to clopidogrel treatment has been attributed to genetic factors. In particular, five SNPs of rs4244285, rs4986893, rs12248560, rs662 and rs1045642 have been associated with resistance to clopidogrel therapy in Chinese population. This work involves the development of a multiplex high-resolution melting (HRM) assay to genotype all five of these loci in 2 tubes. Amplicons corresponding to distinct SNPs in a common tube were designed with the aid of uMelt prediction software to have different melting temperatures Tm by addition of a GC-rich tail to the 5′ end of the certain primers. Two kinds of commercial methods, Digital Fluorescence Molecular Hybridization (DFMH) and Sanger sequencing, were used as a control. Three hundred sixteen DFMH pretested samples from consecutive acute coronary syndrome patients were used for a blinded study of multiplex HRM. The sensitivity of HRM was 100% and the specificity was 99.93% reflecting detection of variants other than the known resistance SNPs. Multiplex HRM is an effective closed-tube, highly accurate, fast, and inexpensive method for genotyping the 5 clopidogrel resistance associated SNPs.

## Introduction

Clopidogrel is widely used in antiplatelet therapy in patients undergoing percutaneous coronary intervention (PCI) to reduce ischemic complications^1^. But the therapeutic efficacy varies significantly^2^. Nearly 22% to 43% patients have low response to clopidogrel^3^, and a higher risk of early cardiovascular events suggesting clinical resistance^4^. Clopidogrel is a prodrug without antiplatelet effect before bioconversion^5^. The intestinal absorption of clopidogrel is regulated by P-gp, a transmembrane protein encoded by the multidrug resistance gene ABCB1^6^. After absorption, clopidogrel is converted to 2-oxo-clopidogrel, which is catalyzed by CYP2C19. In the final step, 2-oxo-clopidogrel is transformed to the active form through the effect of PON1^7^.

Five SNPs that occur in the Chinese population, CYP2C19*2 (rs4244285, G > A), CYP2C19*3 (rs4986893, G > A), CYP2C19*17 (rs12248560, C > T), PON1 (rs662, G > A) and ABCB1 (rs1045642, C > T) have been associated with effects on clopidogrel therapy^8^. Among them, rs4244285A, rs4986893A, rs662A and rs1045642T are loss-of-function variants and rs12248560T is gain-of-function variant. Genotyping assays for these five SNPs associated with clopidogrel treatment are important for personalized decisions regarding clopidogrel therapy.

Various techniques have been used to genotype clopidogrel resistance associated SNP alleles. Sanger sequencing is considered as the gold standard^9,10^. It has a high cost and requires at least one day after PCR to obtain the result. PCR- restriction fragment length polymorphism (PCR-RFLP) genotyping uses restriction enzymes to digest the PCR product for analysis by gel electrophoresis. This is a less expensive than Sanger sequencing for clopidogrel resistance SNP genotyping, but it involves several steps and some are open tube and susceptible to contamination^11^. Allele- Specific PCR (AS-PCR) has also been used for genotyping clopidogrel resistance associated SNPs^12^. Allele-specific primers selectively amplify the target allele, but false positives from non-specific amplification are often observed. Hybridization with fluorescent probes tethered to a gene chip’ has been employed for high-throughput genotyping. The chip price is very high but it can analyze large numbers of samples simultaneously^13^. Denaturing high performance liquid chromatography (DHPLC) has also been used for clopidogrel resistance SNPs genotyping. This approach has the advantage of efficiency with simple automation, but the instrument and the reagent cost is very high^14^. With the development of different kinds of fluorescent hybridization probe, a variety of SNP detection methods based on fluorescent probes have been utilized for clopidogrel resistance SNPs genotyping^15,16^. SPARTAN assay is a kind of point-of-care testing which is based on portable PCR instrument and Taqman fluorescent probe^17,18^. It is closed-tube, fast, flexible used, and only buccal swab is required. But the assay is single target, the cost of the fluorescently labeled probes is high and the throughput is low because of its small size. Digital fluorescence molecular hybridization (DFMH) is based on molecular beacon. This method is closed-tube and easy in sample pretreatment, but the fluorescently labeled probes is also costly.

High-resolution melting (HRM) is a genotyping technique that follows PCR amplification in the same closed tube in which a saturating double strand DNA dye has been included with the reagents. Acquisition of fluorescence during a subsequent denaturation step produces the melting curve that can be used to perform mutation scanning or genotyping SNPs or other variants^19^. By the present time, most real-time PCR instruments have also provided this HRM functionality. HRM genome scanning and genotyping requires only 5 to 10 minutes after PCR for acquiring and analyzing the melting curves. Currently, HRM has been used to genotype individual clopidogrel resistance SNPs^16,20^. But multiplex HRM was not yet seen in clopidogrel resistance genotyping. Here we have developed multiplex HRM to genotype the five clopidogrel resistance associated SNPs in just two reactions, one triplex and one duplex. This method is convenient, fast, closed-tube, cost efficient and reliably accurate.

## Results

### Multiplex PCR and HRM

**Figure 1:**
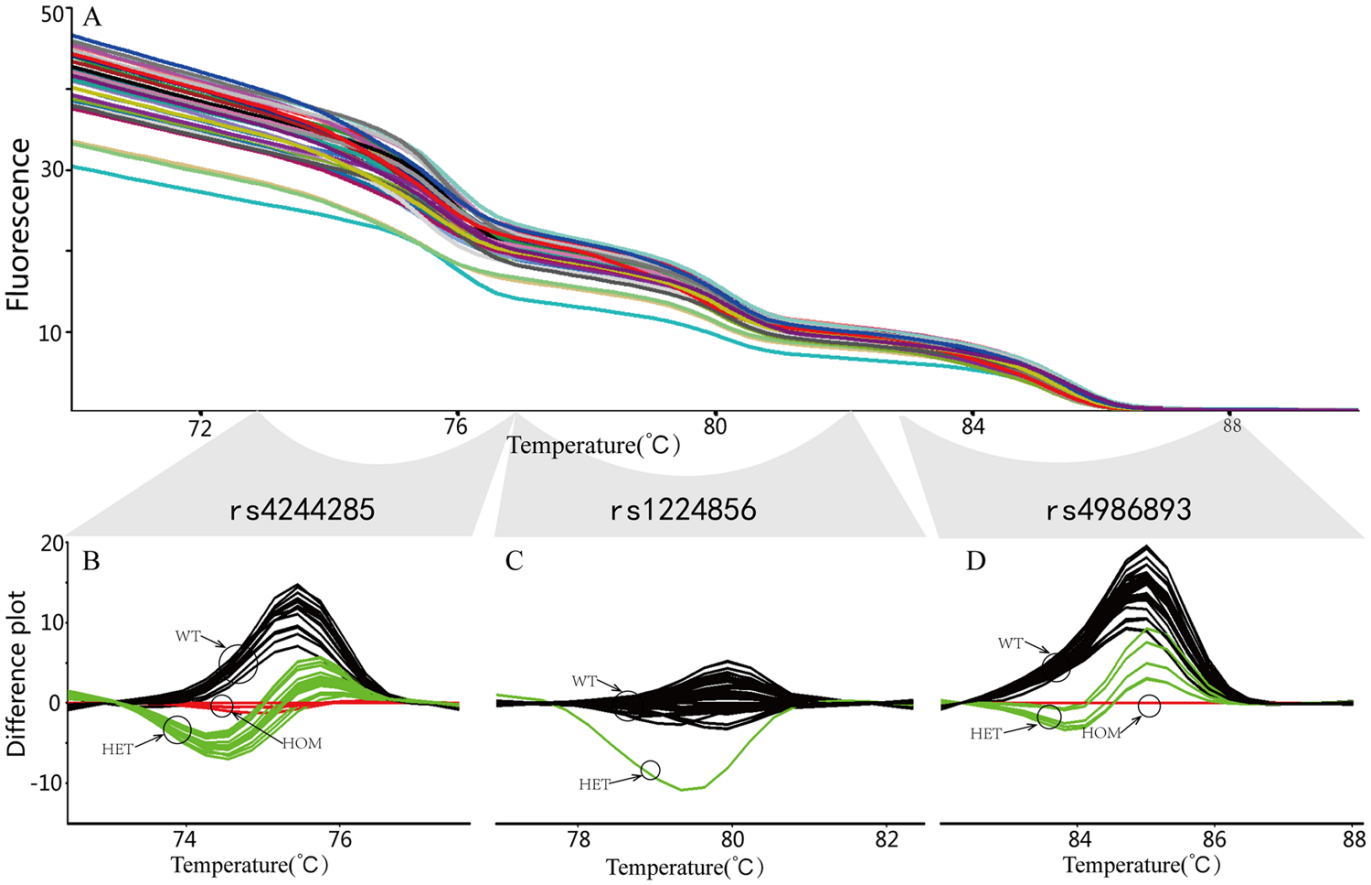
Genotype results of rs662 and rs1045642. (**A**) Raw results of HRM. (**B**) and (**C**) Difference plots of each SNP allele, samples were classified into three clusters, the wild type (WT) samples are black, heterozygous mutation (HET) are green, and homozygous mutant (HOM) are red.

**Figure 2:**
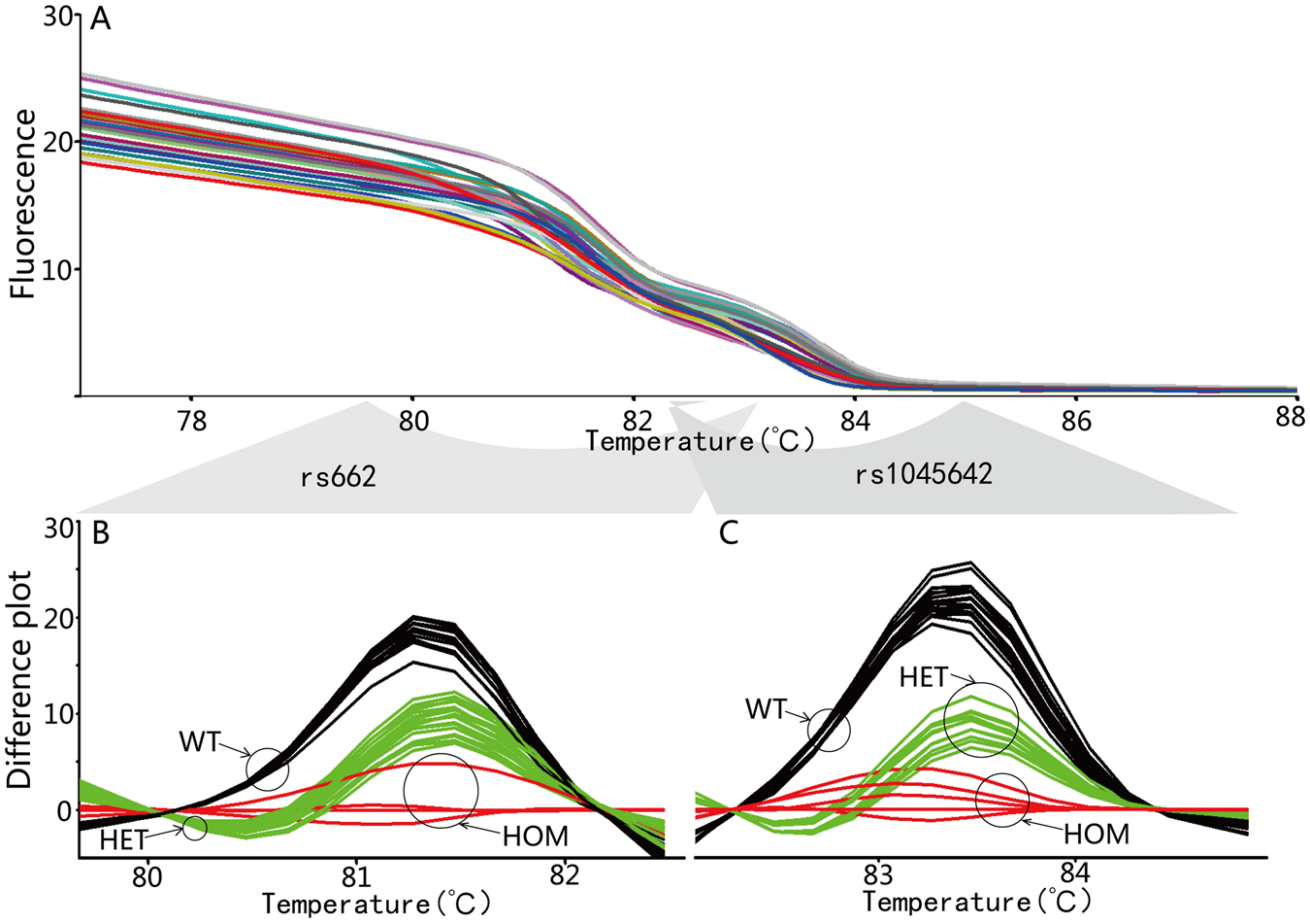
Genotype results of rs4244285, rs4986893 and rs12248560. (**A**) Raw results of HRM. (**B**) to (**D**): Difference plots of each SNP allele. Samples were classified into three clusters, the wild type (WT) samples are black, heterozygous mutant (HET) are green, and homozygous (HOM) mutant are red.

The 5 clopidogrel resistance associated SNPs were amplified in duplex and triplex PCR and genotyped by duplex and triplex HRM. The Tm differences between neighboring amplicon melting transitions in the multiplex HRM were from 2–5 °C. The GC-rich tail was added on 5′ end primers of SNP rs4986893 to obtain this minimum separation by increasing its amplicon Tm in the triplex HRM and isolating the melting region of each SNP in the multiplex HRM (Figs 1A, 2A). Rotor-gene Q Series Software difference plots clearly distinguished the melting curves of wild-type, heterozygous and homozygous mutant genotypes in the transition interval associated with each SNP. (Figs 1B,C,D and 2B,C).

### Amount of DNA of genotyping by multiplex HRM

The concentrations of DNA extracted from patients’ blood in the study varied from 22 to 259 ng/μL and genotyping was robust across the entire range of concentrations.

### Blind test

**Figure 3:**
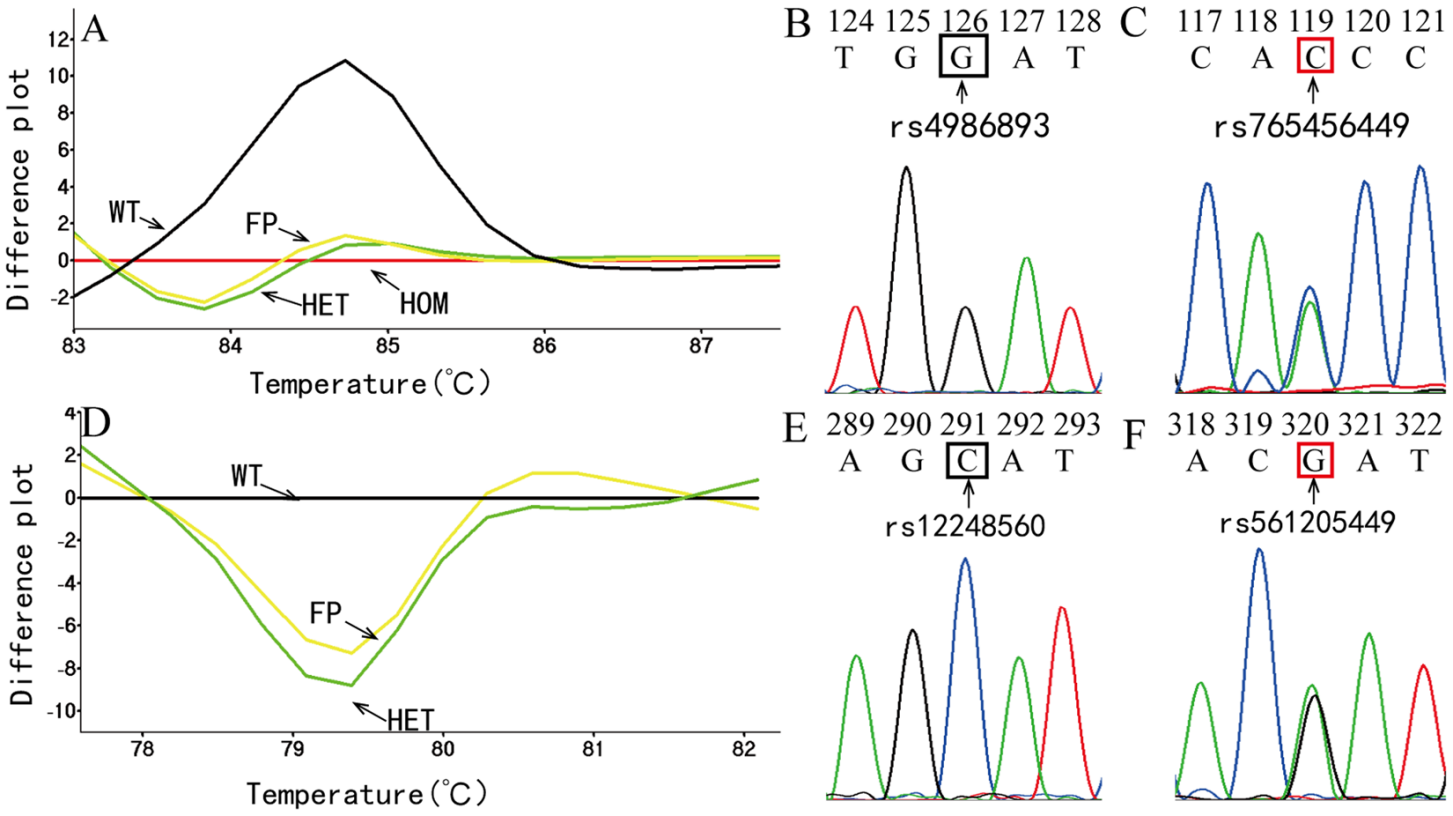
False positive genotyped samples by HRM assay. (**A**) The difference curves of the false positive for rs4986893 sample (yellow, FP), wild type sample (black, WT), heterozygous sample (green, HET), homozygous mutant sample (red, HOM). (**B**) Sanger sequencing results for the target SNP allele of rs4986893 HRM genotyping (green in **A**), and (**C**), sequencing results for the false positive sample (yellow in **A**) showing an alternate variant allele. (**D**) The difference curves of the false positive for rs12248560 sample (yellow, FP), wild type sample (black, WT), heterozygous sample (green, HET). (**E**) Sanger sequencing results for the target SNP allele of rs12248560 HRM genotyping (green in **D**), and (**F**), sequencing results for the false positive sample (yellow in **D**) showing an alternate variant allele.

Samples from 912 patients whose 5 clopidogrel associated SNPs had been previously genotyped by DFMH pretested were used for a blinded test of the multiplex HRM assay. SNP rs662 and rs1045642 were genotyped in duplex HRM (Fig. 1A). SNP rs4244285, rs4986893 and rs12248560 were genotyped in triplex HRM (Fig. 1B). All but 11 samples were genotyped concordantly according to the DFMH and multiplex HRM assays, this was considered confirmation of accuracy for both sets of 5 genotypes. Sanger sequencing was used to determine the genotypes of 11 discordant samples, and in each case the result was concordant with that of one of the two methods. Two of the 11 discrepancies consisted of samples that DFMH identified as wild-type, but HRM identified both as heterozygous variants. Looking more closely, sequencing revealed that both samples did have variants in bases other than the particular SNPs being typed, though whether these variants have any bearing on clopidogrel therapy is beyond the realm of this study. In particular, Sanger sequencing showed that both patient samples were indeed wild-type for the two SNPs rs4986993 G (Fig. 3B) and rs12248560 C (Fig. 3E) associated with clopidogrel therapy, in agreement with DFMH. But one of these that was called heterozygous G/A for rs4986993 by HRM (Fig. 3A) was instead heterozygous A/C for rs765456449 in the same amplicon as rs4986993 (Fig. 3C). The other was called heterozygous C/T for rs12248560 (Fig. 3D) but was actually heterozygous A/G for rs561205449 in the same amplicon as rs4986993 (Fig. 3F). So the only two miscalls according to HRM were both due to detection of alternate heterozygous variants in the amplicons of interest.

In the remaining 9 cases, sequencing and HRM were in agreement, but DFMH results were different. Of these, 7 were false positives and 2 were false negatives (Table 1). One patient was genotyped heterozygous mutation A/G on rs4244285 by DFMH, the sequence showed wild-type A (false positive). Six patients’ genotyped heterozygous mutation G/A on rs4986893 by DFMH, the sequence showed they were wild-type G (false positive). Two patients were genotyped wild-type C on rs1045642 by DFMH, the sequence showed heterozygous mutation C/T (false negative).

**Table 1:** Genotyping results for 5 clopidogrel therapy associated SNPs for 912 patient samples by DFMH and HRM. A total of 9 errors made using DFMH consisted of: (a) Two false negatives on rs1045642 C/T > C/C; (b) One false positive on rs4244285 A/A > A/G; (c) Six false positive on rs4986893 positive G/G > G/A; A total of 2 errors made using HRM: d) One false positive on rs4986893 G/G > G/A; (e) One false positive on rs12248560 C/C > C/T.

	rs4244285		rs4986893		rs12248560		rs662		rs1045642	
	DFMH	HRM	DFMH	HRM	DFMH	HRM	DFMH	HRM	DFMH	HRM
Wild-types	404	405	819	824	907	906	374	374	371^a^	369
Heterozygotes	391^b^	390	91^c^	86^d^	5	6^e^	426	426	422	424
homozygous	117	117	2	2	0	0	116	116	119	119

## Discussion

Various factors contribute to resistance to clopidogrel therapy, including genetic variation, drug-drug interaction, under dosing, body mass index, platelet activation via alternate pathways, and more^21^. Among these, genetic factors are especially important. Accordingly, it is significant to develop an appropriate way to detect SNP alleles related to clopidogrel metabolism before clopidogrel therapy. CYP2C19*2 (rs4244285) is a mutation of exon 5 corresponding to position 681 in the cDNA. Though the genetic codon obtained through the mutation represents the same amino acid, the variation from G to A produces a cryptic splice site in the exon, which shows a similar degree of homology with the mammalian 3′-splice site consensus sequence as with the wild-type sequence, so that a nearly 40-bp deletion can occur in cDNA, resulting in a truncated, non-functional protein^22^. D. Sibbing *et al*.^23^ proved that at least one CYP2C19*2 allele can result in higher platelet aggregation values than for homozygous wild-type. Jessica *et al*.^24^ explored 335 patients with stable cardiovascular disease, and the results showed that the presence of rs4244285 heterozygotes indicates the need for tripling the dose of clopidogrel for noncarriers, yet even doses of 300 mg daily of rs4244285 homozygotes were not able to result in comparable degrees of platelet inhibition as from 75 mg for noncarriers’. The variation of G to A in CYP2C19*3 (rs4986893) creates a premature stop codon, so that there is lack of enzyme activation required for metabolizing clopidogrel^25^. Hong-Guang Xie^26^ reported that the frequency of rs4986893 in the Chinese population is much higher than in the Blacks and Caucasian populations. Other research shows that rs4986893 is considered to be a significant risk factor for clopidogrel resistance of Asian populations^27^. Among the five associated genetic variants, rs12248560 is the only one associated with a gain-of-function. The mutation from C to T in the 5′-flanking region of the gene leads to an increase in transcription of CYP2C19. Researchers found that rs12248560 has a significant impact on ADP-induced platelet aggregation, confers an increased risk of bleeding events, and does not have a protective effect on the occurrence of ST or other ischemic events in clopidogrel-treated patients^28^. SNP rs662 is a mutation from G to A which makes a replacement from Arg to Gln at amino acid position 192. This variation changes activity of paraoxonase-1^29^. Bouman reported that compared to G/G homozygous individuals, A/A homozygous individuals showed a considerably higher risk of stent thrombosis, lower PON1 plasma activity, lower plasma concentrations of active metabolite and lower platelet inhibition^30^. Variation of C to T in rs1045642 is a silent mutation. This polymorphism does not change the amino acid sequence, but results in variation in its expression level^31^. Hoffmeyer *et al*.^32^ investigated this variant in 112 healthy Caucasian volunteers, for whom the results showed that homozygous TT individuals had lower levels of P-gp expression, approximately 2-fold lower compared with homozygous CC individuals., while heterozygous individuals had intermediate expression levels. Taubert *et al*.^6^. reported that peak plasma concentration and total area under the plasma concentration–time curve of clopidogrel and its active metabolite were lower in homozygous T/T patients than heterozygous or homozygous C/C patients after a single oral loading dose of 300, 600 mg.

The HRM technique has been widely used for genome scanning and genotyping. It is a closed tube method that only require a fluorescence vs. temperature melting analysis after PCR. For unknown mutation or SNPs, HRM can be used to scan the target fragment for any variants. If the resulting melting curve is different than wild-type, Sanger sequencing can be used to identify the specific mutation. For well-known mutations, unlabeled probes, snapback primers or short amplicons have been used to genotype the point mutation or SNP by HRM without Sanger sequencing^33^. For amplicons shorter than 200 bp, the heterozygous genotyping sensitivity and specificity is 100% and the homozygous high-resolution melting curve of A/A (or T/T) vs C/C (or G/G) are also clearly distinguishable^34^. According to nearest-neighbor theory, the homozygous high-resolution melting curves of A/A vs T/T and C/C vs G/G when surrounded by complementary bases are indistinguishable, but are sufficiently separated when the surrounding bases are not complementary^35^. Fortunately, all 5 clopidogrel resistance associated SNPs are A/G, G/A and C/T, the longest amplicon designed in the study was only 107 bp, and therefore all 5 SNP homozygous mutant samples are clearly separated from wild-type by HRM.

**Figure 4:**
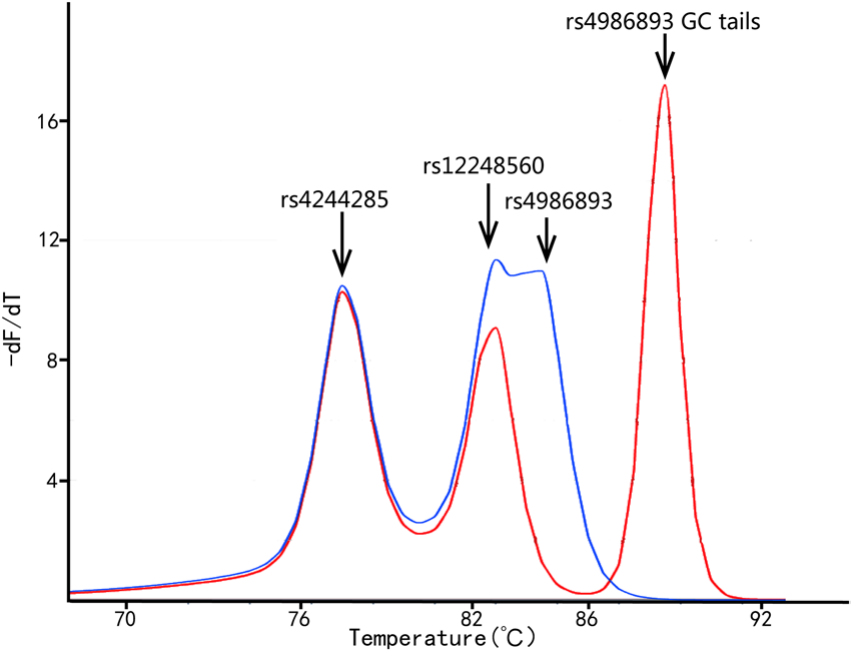
uMelt software Tm prediction of triplex melting peaks with and without GC-rich tails on rs12248560 primers added on 5′ end of rs12248560 primers. The purple curve was amplified by rs4986893 primers without 5′ GC-rich tails and rs12248560 primers. The yellow curve was amplified by rs4986893 primers with 5′ GC-rich tails and rs12248560 primers. The ΔTm increased 3.5 °C with the GC-rich tail added on rs4986893.

Amplicon melting temperature depends in part on amplicon length and GC content. Amplicons having greater length and higher GC content typically have higher melting temperature, although sequence specific effects are also involved. These considerations determine the design of amplicons that could amplify in the same tube and melt within distinct temperature intervals to permit genotyping by multiplex HRM. Genotyping by multiplex HRM was used on clinical targets^36–38^. uMelt software was used as a tool to predict multiplex melting temperatures. In the initial triplex PCR design, the ΔTm between rs4986893 and rs12248560 was only 1 °C. Increasing the length of the amplicon simultaneously increases the likelihood of unexpected variants becoming involved and causing false positive calls. Therefore, a GC rich tail was added instead on the end of rs4986893 primers instead as a means to increase the melting temperature separation. The GC rich tail on rs4986893 increased the amplicon melting temperature 3.5 °C, increasing the ΔTm between rs4986893 and rs12248560 to 4.5 °C (Fig. 4), so that the melting behavior of both SNPs rs4986893 and rs12248560 could be easily distinguished and genotyped. The amplicon Tm differences predicted by the uMeltBatch software (https://www.dna.utah.edu/umelt/umb.php) were sufficiently accurate, although the individual Tms differed from experiment in part due to the fact the dye used in the study was Syto9 while the software is based on the LCGreen Plus dye.

Two false positive genotype calls were made by HRM. Both of these were based on melting curves that appeared similar to those of samples having the heterozygous mutation. Sequencing confirmed that the respective targets rs4986893 and rs12248560 were wild-type. The heterozygous melting curve shapes in these amplicon regions were caused by nearby heterozygous SNPs rs765456449 and rs561205449. High-resolution melting analysis uses melting curve changes to determine the nucleotide change. For every sample having only variation in the intended target in the amplicon, the melting curve correctly identifies the genotype. In those rare cases in which another SNP is present in the amplicon, the resulting melting curve change may not be distinguishable from the change due to the expected variant^33,34^. For this reason, it is useful to make the amplicon as short as possible to avoid alternate variants as much as possible. In this study, even with relatively short amplicon design, 2 such variants were observes, and the heterozygous melting curves of the alternate variant SNPs rs765456449 (A/C) and rs561205449 (A/G) were insufficiently distinguishable from those of the corresponding intended target heterozygous mutations rs4986893 (A/G) and rs12248560 (C/T), respectively (Fig. 3A,D). The multiplex HRM genotyping sensitivity was 1328/1328 = 100% and specificity due to these 2 false positives was 2882/2884 = 99.93%. These impostor mutations are quite rare in Chinese population. The frequency of the minor allele of rs765456449 is only 0.00002471 in whole population, and the minor allele of rs561205449 is 0.0002 in the same range. The study shows that multiplex HRM has extremely high accuracy for genotyping all 5 clopidogrel resistant SNP, in addition to the many advantages of cost, speed, simplicity, and low susceptibility to contamination. The accuracy of multiplex HRM genotyping was observed to be robust in a wide range of template DNA concentrations from 22 ng/µL to 259 ng/µL.

The DFMH method for genotyping the 5 clopidogrel resistant SNP used for comparison in this study uses an unpublished commercial kit. DFMH genotyping uses hybridization of molecular beacon probes labeled with FAM and ROX for each SNP’s two alleles to the template, without PCR amplification. After several cycles of denaturing at 95 **°**C and hybridizing at 55**°**C, if the probe matches the template allele, the FAM or HEX fluorescence will be detected at its hybridization temperature. If only FAM or HEX signal is observed, it indicates that the sample is homozygous wild-type or mutant, respectively. If both FAM and HEX signals are observed, it indicates that the sample is heterozygous. Because no PCR is involved in the DFMH genotyping process, the DNA extraction kit can be very crude (see method), because clean template DNA is not required. While DFMH is therefore a fast technique, 9 of the in 11 discordant samples were due to errors made by DFMH. Sanger sequencing confirmed that DFMH had 2 false negatives and 7 false positive. The DFMH sensitivity was 1683/1685 = 99.88% and specificity was 2882/2889 = 99.76%. DFMH genotyping for clopidogrel resistant SNP is based on a commercial kit. The DFMH technique is based on FAM and HEX probes hybridized to the unamplified template. Because the hybridization fluorescence signal was not sufficient to be detected with one acquisition, and therefore the fluorescent hybridization signal is acquired digitally multiple (n = 55) times, and the collective results processed using the commercial DFMH software. The details of the technique are unpublished, and the specifics of the probes, reagents and the principles of the software could not be found. Genotyping error made by DFMH could likely be caused by genomic DNA affecting probe hybridization or the strength and accuracy of the signal. Advantages of the DFMH method include that it genotypes without PCR amplification, so that there is no PCR related contamination and the DNA samples do not need to be as high quality as needed for PCR. Disadvantage of DFMH included the fact that DNA prepared from DFMH kit was too crude, with red blood cells remaining in the extracted DNA solution. Combining a DFMH kit with an alternate DNA extraction could enhance the DFMH results by providing a stronger hybridization signal. The DFMH process requires almost 2 hours to complete, much longer than multiplex PCR and HRM. DFMH is specific to a particular platform, TL998A real-time thermocycler, and requires fluorescently labeled probes, in contrast to multiplex HRM.

Multiplex HRM is cheaper, faster and more accurate than the commercial DFMH kit. In the future, we aim to further simplify these assays to be able to genotype these 5 SNPs in just one set of PCR conditions.

In conclusion, multiplex HRM is a rapid, high-throughput, convenient, inexpensive and reliable method that is suitable for diagnostic assays of clopidogrel resistance related 5 SNPs.

## Materials and Methods

### Ethics statement

Informed consent was obtained from all subjects. All experimental protocols were approved by the Ethics Committee of the Shanghai Children’s Medical Center and Renji Hospital and carried out in accordance with relevant guidelines.

### DNA Samples

This study was based on samples obtained from 912 consecutive acute coronary syndromes patients undergoing PCI, and receiving dual antiplatelet therapy including clopidogrel and aspirin, comprised of 213 women and 699 men with age between 35–94 years. All patients’ whole blood samples were obtained from Renji hospital with EDTA as anticoagulant.

Genomic DNA was extracted from 500 uL whole blood by automatic DNA extraction system Lab-Aid 820 (Zeesan Biotech Co., Xiamen, China). The extracted DNA was quantified using Nanodrop 2000 (Thermo, Waltham, US). The concentration of DNA samples ranged from 22 to 259 ng/μL.

### Primers

The Primer3 (v.0.4.0) online software (http://bioinfo.ut.ee/primer3–0.4.0/primer3/) was used to design primers for the five SNP loci. The uMELT online software (https://www.dna.utah.edu/umelt/umelt.html) was used to predict amplicons’ melting temperature. The primer sequences used to amplify the regions surrounding the clopidogrel resistance associated SNPs are shown in Table 2. The bases forming the extra GC-rich tail added to adjust and distinguish the melting temperature on 5′ end of rs4986893 primers are underlined in Table 2. The primers were synthetized by Sangon Biotech (Shanghai, China).

**Table 2:** Primers sequences for the multiplex HRM PCR amplification. The underlined bases belong to the GC-rich tail.

Gene	Code	Primers	Length (bp)	Tm (°C)
CYP2C19*2	rs4244285	CCCACTATCATTGATTATTTCC	81	75.8
		CTCCAAAATATCACTTTCCATA		
CYP2C19*3	rs4986893	CCGGCGGCGGAAAACATCAGGATTGTAAGC	100	85.3
		CGGCGGCCGCAAAGACTGTAAGTGGTTTCTCA		
CYP2C19*17	rs12248560	AACAAAGTTTTAGCAAACGATTT	106	80
		TGAGGTCTTCTGATGCCCAT		
PON1	rs662	TTGTTGCTGTGGGACCTG	103	81.7
		TACGACCACGCTAAACCC		
ABCB1	rs1045642	GGGTGGTGTCACAGGAAGA	107	83.9
		CCCAGGCTGTTTATTTGAAG		

### PCR and HRM Assay

Both the PCR and HRM steps were performed on the Rotor-gene Q (Qiagen, Duesseldorf, German). PCR was performed in 20 μL volumes containing 1.5 mmol/L Mg^2+^, 200 μM of each dNTP, 0.5 U of rTaq polymerase (Takara, Tokyo, Japan), 50 pmol Syto9 (Invitrogen, Waltham, US) and genomic DNA range was from 22 ng to 259 ng. The primer concentrations were optimized individually for the specific SNP amplicons. In the duplex PCR, the concentration of each rs662 primer was 0.15 μM, and 0.35 μM of each rs1045642 primer. In the triplex PCR, the concertation of each rs4244285 primer was 0.35 μM, 0.1 μM of each rs4986893 primer, and 0.35 μM of each rs12248560 primer. The PCR amplification protocol began with an initial denaturation at 95 °C for 3 minutes, followed by 35 cycles of 15 seconds denaturation at 95 °C and 30 seconds annealing-extension at either 62 °C (for the duplex PCR) or 60 °C (for triplex PCR). After amplification, the samples were cooled to 55 °C then melted from 55 °C to 95 °C at the melting rate of 0.2 °C/second. The melting curves were analyzed by Rotor-gene Q Series Software (Qiagen, Duesseldorf, German). Standard genotype reference samples of each genotype in each SNP sites were confirmed by Sanger Sequencing. The sample genotypes were determined by greatest similarity of their melting curves to those of standard genotype reference samples.

### DFMH

For the purpose of comparing and validation, clopidogrel resistance SNPs genotyping by DFMH (which has been widely used in China) was also performed using a commercial kit from Sino Era Genotech (http://www.sino-era.com/, Beijing, China). The kit includes reagents for DNA extraction, molecular beacon probes and hybridization buffer. 200 μL of whole blood was added to 1 mL of red blood cell lysis buffer and incubated for 5 minutes at room temperature. The mixture was centrifuged (3000 rpm) for 5 minutes and the supernatant removed. 50 μL preservation solution was added to resuspend the precipitate. 1.5 μL of sample was then combined with DFMH detecting reagent and DFMH analysis was then performed with the TL998A real-time PCR system (Tianlong, Xi’an, China). The detecting reagent includes hybridization buffer and two molecular beacon probes labeled with FAM and HEX for wild-type and mutation, respectively. Five different detection reagents in 5 tubes were used to genotype the five targets. The detection process began with denaturation for 5 minutes at 95 °C followed by 55 cycles of denaturation for 30 second at 95 °C and hybridization for 75 seconds at 62 °C. Though this process is similar to PCR, no amplification occurs as there are no primers in the reaction. DFMH detection is based on sample template alone.

### Sanger Sequencing

The primers for Sanger sequencing are shown in Table 3. Sequencing was performed by fluorescent dideoxy-nucleotide termination on the ABI Prism 3730xl DNA Sequencer (Applied Biosystems, USA) at Map Biotech (Shanghai, China). The sequence data were analyzed with Bioedit V7.0.9.0.

**Table 3:** Primers sequences for Sanger sequencing.

Gene	Code	Primers
CYP2C19*2	rs4244285	AAAGCAGGTATAAGTCTAGGAAATG
		ATAAAGTCCCGAGGGTTGTT
CYP2C19*3	rs4986893	CAATCATTTAGCTTCACCCTG
		TTGGGATATTCATTTCCTGTG
CYP2C19*17	rs12248560	GCCCTTAGCACCAAATTCTCT
		CACCTTTACCATTTAACCCCC
PON1	rs662	GGATTGTATCGGCAGGAC
		ACTTGCCATCGGGTGA
ABCB1	rs1045642	GGCAAAGAAATAAAGCGACTG
		GGTAAAGGTAACAACTAACCCAAAC
